# The construction and optimization of engineered yeast chassis for efficient biosynthesis of 8‐hydroxygeraniol

**DOI:** 10.1002/mlf2.12099

**Published:** 2023-12-26

**Authors:** Yu Zhang, Mengdi Yuan, Xinxin Wu, Qiuhui Zhang, Yuzhu Wang, Liming Zheng, Tsan‐Yu Chiu, Huiming Zhang, Lei Lan, Feng Wang, Ying Liao, Xuemei Gong, Shirui Yan, Yun Wang, Yue Shen, Xian Fu

**Affiliations:** ^1^ BGI Research Shenzhen China; ^2^ BGI Research Hangzhou China; ^3^ Guangdong Provincial Key Laboratory of Genome Read and Write, BGI Research Shenzhen China; ^4^ BGI Research Changzhou China

**Keywords:** 8‐hydroxygeraniol biosynthesis, G8H, SCRaMbLE, synthetic yeast

## Abstract

Microbial production of monoterpenoid indole alkaloids (MIAs) provides a sustainable and eco‐friendly means to obtain compounds with high pharmaceutical values. However, efficient biosynthesis of MIAs in heterologous microorganisms is hindered due to low supply of key precursors such as geraniol and its derivative 8‐hydroxygeraniol catalyzed by geraniol 8‐hydroxylase (G8H). In this study, we developed a facile evolution platform to screen strains with improved yield of geraniol by using the SCRaMbLE system embedded in the Sc2.0 synthetic yeast and confirmed the causal role of relevant genomic targets. Through genome mining, we identified several G8H enzymes that perform much better than the commonly used CrG8H for 8‐hydroxygeraniol production in vivo. We further showed that the N‐terminus of these G8H enzymes plays an important role in cellular activity by swapping experiments. Finally, the combination of the engineered chassis, optimized biosynthesis pathway, and utilization of G8H led to the final strain with more than 30‐fold improvement in producing 8‐hydroxygeraniol compared with the starting strain. Overall, this study will provide insights into the construction and optimization of yeast cells for efficient biosynthesis of 8‐hydroxygeraniol and its derivatives.

## Impact statement

Efficient production of high‐value natural products such as monoterpenoid indole alkaloids in heterologous microorganisms is an important and impactful research field of synthetic biology, which often requires genomic modifications of the chassis and engineering of introduced enzymes. Taking the advantage of the SCRaMbLE system designed in the Sc2.0 synthetic yeast, we developed a facile screening method to identify strain with improved geraniol production after inducible genome rearrangements. We also demonstrated that genome mining of superior enzyme homologs would be an effective and powerful strategy to overcome the rate‐limiting step. These findings expand the current methods to optimize the biosynthesis pathway of 8‐hydroxygeraniol and its derivatives.

## INTRODUCTION

Monoterpenoid indole alkaloids (MIAs) are a class of plant natural products that include many compounds with high pharmaceutical values such as the famous antitumor drugs vinblastine and vincristine. Although in high demand, vinblastine and vincristine only accumulate in trace amounts in the natural species (approximately tons of dry leaves is needed to obtain gram‐scale vinblastine in *Catharanthus roseus* ^1^). With the rapid development of synthetic biology technologies and increased knowledge about the biosynthetic pathway^2^, microbial production of MIAs using heterologous hosts (such as yeast) serves as an efficient, scalable, and economical way. Recent works^3^, ^4^ have successfully reconstructed the biosynthesis pathway to produce key vinblastine precursors in *Saccharomyces cerevisiae*. In these studies, the introduction of two heterologous enzymes, geraniol synthase (GES) and geraniol 8‐hydroxylase (G8H), into budding yeast allows the formation of 8‐hydroxygeraniol from geraniol using geranyl pyrophosphate (GPP) as the endogenous substrate.

As 8‐hydroxygeraniol is the key precursor of MIAs, its intracellular concentration is closely related to the biosynthesis of MIAs. The deficiency of 8‐hydroxygeraniol in microbial cells is caused by two major factors: (i) low activity of heterologous G8H^3^, ^5^ and (ii) low accumulation of geraniol^4^. The G8H belongs to the cytochrome P450 (CYP) monooxygenase CYP76B family, which was found in MIAs‐producing plants, such as the *Apocynaceae*, *Rubiaceae*, and *Gentianceae* family^6^, ^7^. The one from *C. roseus* (CrG8H) was confirmed to catalyze the hydroxylation of geraniol C8 position in yeast cells but have low activity^3^, ^8^. So far, gene overexpression by increasing the gene copy number serves as a common strategy to overcome low activity of CrG8H enzyme^3^, ^8^, ^9^, ^10^, ^11^. Apart from this, screening for active G8Hs from the MIAs‐producing plants is a promising solution to improve the production of 8‐hydroxygeraniol or MIAs in microbial cells^5^, ^8^. Using the above strategies together with the optimization of fermentation conditions, previous studies have achieved the biosynthesis of different levels of 8‐hydroxygeraniol in yeast cells ranging from mg/l to g/l (Table S1).

Accumulation of sufficient geraniol is also critical for improving the yield of 8‐hydroxygerainol, which often requires metabolic engineering of the biosynthesis pathway. Several strategies have been shown to increase the supply of geraniol, including truncation of GES enzyme or utilization of fusion tag^12^ to improve its heterologous expression^13^, ^14^, engineering of mevalonate (MVA) pathway to enhance GPP concentration^15^, ^16^, and directed evolution of GES to improve its catalytic activity^17^, ^18^. Despites these successes, little efforts focused on systematic optimization of the metabolic network to minimize the incompatibility between the heterologous biosynthesis pathway and the chassis. The current methods for improving the chassis compatibility with heterologous pathways include yeast gene deletion collection screening technology^19^, CRISPR genome editing technology^20^, and genome minimizing technology^21^. With the rapid development of synthetic genomics, the synthetic yeast genome project (Sc2.0) offers exciting opportunities to promote metabolic engineering in a faster and more efficient manner. As the unique built‐in feature of Sc2.0 yeast genome, the SCRaMbLE system enables inducible and quick generation of massive genotypic diversities via genome shuffling^22^. Utilization of the SCRaMbLE system has been successfully applied to increase yield of terpenoid metabolites such as lycopene^23^ and betulinic acid^24^ with identification of new regulatory genes.

In this study, we achieved efficient production of 8‐hydrogeraniol in budding yeast by both engineering of the biosynthetic pathway and chassis optimization. Taking the advantage of the SCRaMbLE system embedded in the Sc2.0 synthetic yeast, we established a rapid evolution platform to screen strains with improved yield of geraniol. We also confirmed the causal role of several SCRaMbLE‐mediated genomic targets involved in the enhancement of geraniol yield. To increase the conversion of geraniol to 8‐hydroxygeraniol, one of the key rate‐limiting steps during MIAs biosynthesis, we identified several G8H enzymes that are much more active than the commonly used CrG8H via bioinformatic analysis and determined the essential role of its N‐terminus of G8H that affects 8‐hydroxygeraniol production in vivo. Overall, this study could provide guidance for the efficient synthesis of 8‐hydroxygeraniol in yeast and lay the foundation for future industrial production of MIA derivatives.

## RESULTS

### Engineering of 8‐hydroxygeraniol biosynthesis pathway

**Figure 1:**
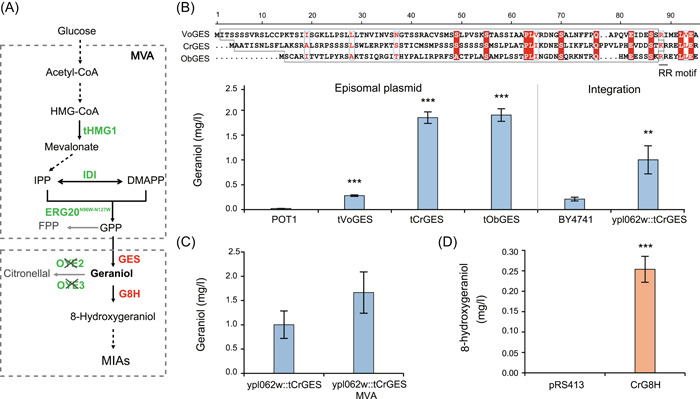
Construction and optimization of 8‐hydroxygeraniol‐producing strains. (A) Schematic diagram of biosynthetic pathway of monoterpenoid indole alkaloid (MIA) precursor 8‐hydroxygeraniol in *Saccharomyces cerevisiae*. The enzymes in red denote the heterologous enzymes from plants, those in green denote the native yeast enzymes, and the cross indicates the deactivation. The solid arrows represent a single enzymatic conversion, whereas dashed arrows indicate multiple enzymatic steps. Gray arrows indicate downregulated step. (B) The optimization of geraniol yield by engineering heterologous synthetic enzyme geraniol synthase (GES). The above panel shows multiple sequence alignment of GESs from three different plant sources: *Valeriana officinalis* (VoGES), *Catharanthus roseus* (CrGES), and *Ocimum basilum* (ObGES). Amino acids with high conservation are indicated by red boxes. The truncated plastid targeting sequence (PTS) amino acids of GESs before RR motif region are in frame, VoGES (2–87 aa), CrGES (2–87 aa), and ObGES (2–72 aa). The left panel shows the geraniol production of optimizing heterologous GES (tVoGES, tCrGES, tObGES) on episomal plasmids and empty vector (POT). The right panel shows the geraniol production of tCrGES integration on genome strain (ypl062w::tCrGES) and wild‐type strain (BY4741). (C) The optimization of geraniol yield by engineering yeast endogenous mevalonate (MVA) pathway. ypl062w::tCrGES MVA strain with three genes of the MVA pathway overexpressed compared to ypl062w::tCrGES strain. (D) The production of 8‐hydroxygeraniol after introduction of heterologous enzyme geraniol 8‐hydroxylase from *C. roseus* (CrG8H). CrG8H and pRS413 represent ypl062w::tCrGES MVA strain expressing CrG8H on the pRS413‐based plasmid and empty vector, respectively. The geraniol and 8‐hydroxygeraniol yield in fermentation culture of strains was determined using gas chromatography–mass spectrometry (GC‐MS). Error bars show the mean ± standard error of three biological replicates. Student's two‐tailed *t*‐test was used for significance test (**p < 0.01, ***p < 0.001).

Biosynthesis of 8‐hydroxygeraniol in budding yeast requires introduction of two heterologous enzymes, GES and G8H (Figure 1A). We first chose GES from *Valeriana officinalis* (VoGES), *Catharanthus roseus* (CrGES), and *Ocimum basilum* (ObGES) in this study according to previous studies^14^, ^25^, ^26^. As the N‐terminal plastidial transit peptide (PTS) present in plant monoterpene synthase (such as GES) may lead to abnormal location of the enzyme in yeast cells, this sequence motif is often removed in investigating heterologous biosynthesis pathways^13^. Previous work also identified that the conserved N‐terminal tandem pair of arginines (RR motif) could be used as truncation position^27^. Thus, we removed the N‐terminal motif sequence of GESs after alignment (Figure 1B), resulting in the truncated GES variants called tVoGES, tCrGES, and tObGES. The yeast cell transformed with plasmid expressing tCrGES and tObGES produced geraniol sevenfold increase compared with tVoGES (Figure 1B), suggesting these two GESs were more efficient for GES. We then integrated the gene encoding tCrGES into the genome of the wild‐type strain (BY4741) to obtain strain ypl062w::tCrGES that enables the production of geraniol yield at 1.00 mg/l (Figure 1B). For further optimization, three known targets of the MVA pathway on upstream of the GES catalytic steps, including *tHMG1* (truncated HMG‐CoA reductase), *IDI* (IPP isomerase), and *ERG20* ^N96W‐N127W^ (geranyl diphosphate synthase mutant) genes^25^, were overexpressed on *GAL80* gene locus, resulting in an improved strain called ypl062w::tCrGES MVA with further 1.66‐fold increase in geraniol biosynthesis (Figure 1C). Finally, we introduced the second heterologous enzyme, G8H from *Catharanthus roseus* (CrG8H), into the ypl062w::tCrGES MVA strain that could produce 0.25 mg/l of 8‐hydrogeraniol (Figure 1D). This observation was encouraging as the strain only expressing tVoGES and CrG8H could not produce detectable amount of 8‐hydroxygeraniol (data not shown). Taken together, we successfully established the 8‐hydroxygeraniol biosynthesis pathway in *S. cerevisiae* via multistep optimizations.

### Screening of yeast cells with improved geraniol production via SCRaMbLE

**Figure 2:**
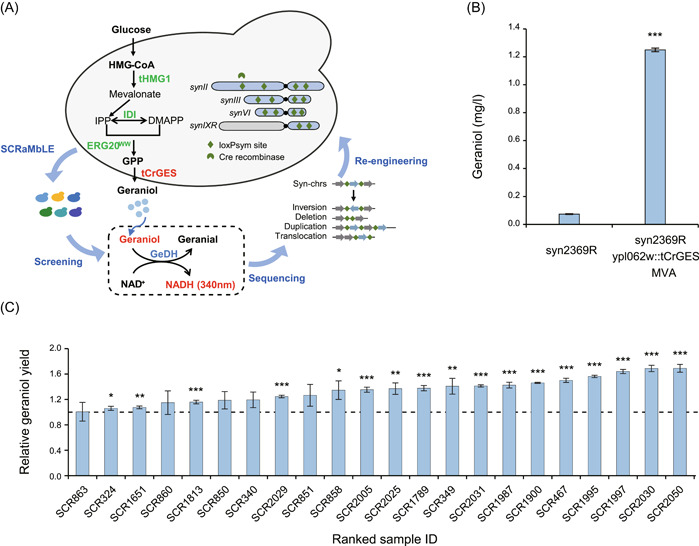
Improving the geraniol yield in synthetic yeast chassis by genome‐directed evolution via SCRaMbLE. (A) The workflow of SCRaMbLE for improving the geraniol production in synthetic strains. Upon estradiol‐induced SCRaMbLE, Cre enzyme cleaved and recombined loxPsym sites. First, SCRaMbLE was conducted to generate a large pool of genotype‐diverse yeasts. Then, enzymatic reaction in vitro was used for screening SCRaMbLEd stains, detecting NADH generated from geraniol redox at 340 nm. Next, whole genome sequencing (WGS) was used to analyze genome structural variations in high‐yield strains. Finally, geraniol‐promoting genes could be reengineered in chassis. (B) Comparison of geraniol production between parental and modified synthetic yeast strains used for SCRaMbLE. syn2369R: synthetic yeast strain carrying multiple synthetic chromosomes (*synII*, *synIII*, *synVI*, and *synIXR*); syn2369R ypl062w::tCrGES MVA strain: syn2369R strain with different genomic modifications including gene encoding tCrGES integrated at *ypl062w* loci and optimized MVA pathway as shown in Figure 1. (C) The screening of geraniol high‐yield SCRaMbLEd strains by detecting fermentation product. The geraniol yield in fermentation culture of strains was determined using GC‐MS. The relative geraniol yield is geraniol yield of SCRaMbLEd strain/geraniol yield of starting strain. Error bars show the mean ± standard error of three biological replicates. Student's two‐tailed *t*‐test was used for significance test (*p < 0.05, **p < 0.01, ***p < 0.001).

Despite our success in the heterologous production of 8‐hydroxygeraniol in yeast cells, its yield is quite low. As proposed in previous studies^4^, deficiency of the precursor geraniol might lead to the low production of 8‐hydroxygeraniol. To repurpose the yeast metabolism for improved geraniol production, we designed a SCRaMbLE‐mediated selection workflow using a Sc2.0 yeast strain that harbors multiple synthetic chromosomes (syn2369R:*synII*, *synIII*, *synVI*, and *synIXR*) with a total of 473 loxPsym sites (Figure 2A). As described above, we constructed the starting haploid strain syn2369R ypl062w::tCrGES MVA that allows the production of 1.25 mg/l geraniol (Figure 2B).

Due to the colorless nature of geraniol, it is difficult to rapidly screen the high‐yield geraniol strains after SCRaMbLE. To overcome this challenge, we designed an in vitro reaction to quickly determine the concentration of geraniol in the fermentation broth based on the change of OD_340_ value. Specifically, the geraniol is catalyzed by geraniol dehydrogenase (GeDH) accompanied by the reduction of cofactor NAD^+^ to NADH, which could be detected by the microplate reader at the wavelength of 340 nm. To validate the effectiveness of this screening method, we tested the response concentration within 0–8 mg/l geraniol. Our results showed a good correlation between the change of OD_340_ and the concentration of geraniol in both medium and fermentation broth (Figure S1). In addition, we found that the optimal ratio of fermentation broth and enzyme reaction buffer was 1:1 to maintain the optimum pH 8.0 of GeDH enzyme (Figure S2). Next, by utilizing this method, a total of 2160 strains subjected to SCRaMbLE were screened, and 45 out of them were found to exhibit a 1.5‐fold increase in relative ∆OD_340_ compared with the starting strain (Figure S3). To verify whether the yield of geraniol in 45 candidate strains was indeed increased, geraniol from the fermentation products of these strains was measured using gas chromatography–mass spectrometry (GC‐MS). Compared with the starting strain, a total of 22 SCRaMbLEd strains showed yield improvement, among which 19 strains with a 14%–69% increase in geraniol production were obtained (Figure 2C).

### Analysis of various structural variation targets responsible for enhancement of geraniol yield

**Figure 3:**
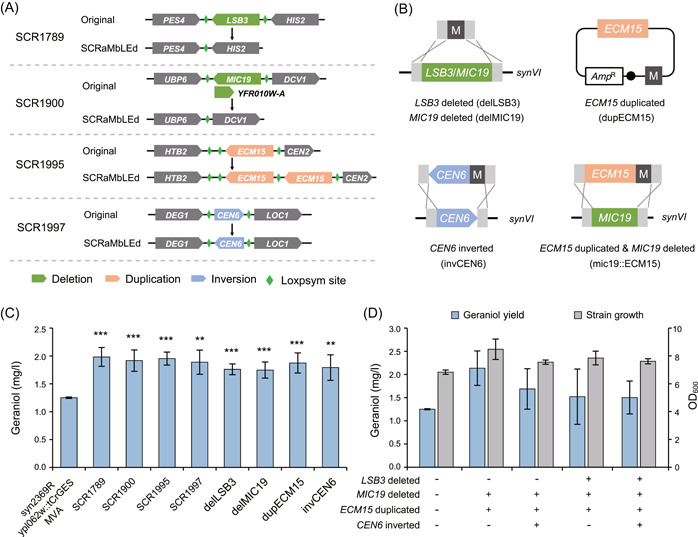
The analysis and verification of genomic variations related to high yield of geraniol. (A) The genome variations observed in four high‐yield geraniol rearranged strains. Green, orange, and blue indicate deletion, duplication, and inversion genes, respectively. Gray indicates upstream or downstream genes of structural variation. (B) Reconstructed strategies of genomic variation targets. The duplication was reconstructed on the episomal plasmid. Deletion, inversion, duplication & deletion were reconstructed on the genome using the yeast homologous recombination method, and the length of the homologous arm was 60 bp. (C) The geraniol yield of SCRaMbLEd strains and reconstructed strains. (D) The geraniol yield and OD_600_ of strains with potential synthesis regulatory targets combined. The blue and gray column represent the geraniol production and the growth of strains after fermentation, respectively. The geraniol yield in fermentation culture of strains was determined using GC‐MS. Error bars show the mean ± standard error of three biological replicates. Student's two‐tailed *t*‐test was used for significance test (**p < 0.01, ***p < 0.001).

We next performed whole genome sequencing (WGS) to analyze the genomic rearrangements that are responsible for the high yield of geraniol. We observed various structural variations in 11 SCRaMbLEd strains (Table S2). Among them, we found four strains harboring simple recombination events just involving one or two genes. SCRaMbLEd strain SCR1789 only contains a 1975 bp deletion, involving the *LSB3* gene that encodes a protein involved in actin cortical patch localization; SCR1900 stain contains two deletions, one of which is *MIC19* gene expressing the component of the mitochondrial inner membrane complex, and the other is *YFR010W‐A* with unknown function. SCR1995 strain contains a tandem repeat of 893 bp, involving the gene *ECM15*, whose function might be related to the yeast wall biogenesis. SCR1997 strain includes only an inversion of *CEN6*, which was also observed in the other two high‐yield geraniol strains, SCR2029 and SCR2031 (Figure 3A and Table S2).

To dissect the causal relationship between the structural variation and the enhanced ability to produce geraniol, we individually reconstructed these genomic variations (*LSB3* deletion, *MIC19* deletion, *ECM15* duplication, and *CEN6* inversion) in the starting strain syn2369R ypl062w::tCrGES MVA using different strategies as shown in Figure 3B. Compared with the starting strain, the reconstructed strains showed a 40%–50% increase in geraniol yield, which was at a similar level to that of the SCRaMbLEd strains (Figure 3C). To determine whether the combination of different recombination events would further enhance geraniol production, we constructed a series of strains with two to four modifications of the targets identified by SCRaMbLE. The resultant strain mic19::ECM15 involving two targets (*MIC19* deletion and *ECM15* duplication) produced geraniol at 2.14 mg/l, whose yield was more than 70% of the starting strain (Figure 3D). However, strains with combinations of three or four targets had adverse effect on the biosynthesis of geraniol (Figure 3D). To further understand this, we performed growth measurement for strains with different number of genomic modifications and found that derivative strains with an additional modification (invCEN6) indeed exhibited growth defect compared with the parental strains (Figure S4), suggesting that combination of more targets does not necessarily play synergistic effect and might cause excessive metabolic burden in the complicated metabolic network. Overall, we disclosed two novel targets, *MIC19* deletion, and *ECM15* duplication, that are beneficial for enhancing geraniol production both individually and collectively.

### Genome mining of G8Hs to enhance 8‐hydroxygeraniol biosynthesis pathway

**Figure 4:**
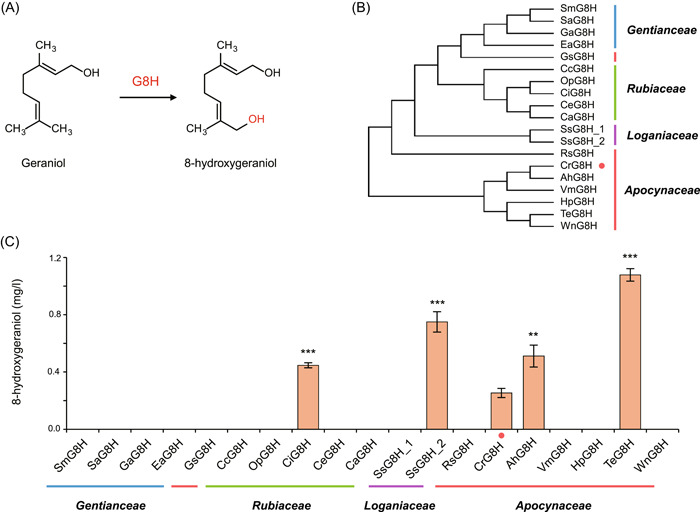
The optimization of rate‐limiting enzyme geraniol 8‐hydroxylase (G8H) on 8‐hydroxygeraniol synthetic pathway. (A) The schematic diagram of conversion reaction where C8 of geraniol was hydroxylated to 8‐hydroxygeraniol by G8H. (B) Phylogenetic tree of 18 kinds of G8H enzymes from MIA‐producing medicinal plants and their source families. Blue, green, purple, and orange indicate G8H from *Gentianceae*, *Rubiaceae*, *Loganiaceae*, and *Apocynaceae*. The dot indicates control CrG8H. (C) The 8‐hydroxygeraniol production in engineered *Saccharomyces cerevisiae* strains harboring G8Hs from different plant sources. The 8‐hydroxygeraniol yield in fermentation culture of strains was determined using GC‐MS. Error bars show the mean ± standard error of three biological replicates. Student's two‐tailed *t*‐test was used for significance test (**p < 0.01, ***p < 0.001).

Hydroxylation of geraniol C8 position to 8‐hydrxygeraniol catalyzed by G8H enzyme is also a rate‐limiting step during the biosynthesis of 8‐hydrogeraniol (Figure 4A). Considering that the low activity of widely used CrG8H would limit the efficient production of 8‐hydrogeraniol in yeast cells^3^, ^5^, we performed genome mining analysis of G8H genes from MIA‐abundant plants (*Apocynaceae*, *Loganiaceae*, *Rubiaceae*, etc*.*) to screen for better enzymes. Using CrG8H sequence as the reference, we identified 18 new G8H genes from the open‐source data from 1000 Plant transcriptomes initiative (1 KP)^28^ and NCBI Nucleotide Sequence Database (NT) (Figure S5 and Table S3). A phylogenetic tree was built by performing multiple sequence alignment using these G8H homologs (Figure 4B). For the purpose of activity comparison, we constructed a series of pRS413‐based plasmids expressing these G8H enzymes and transformed them into the strain ypl062w::tCrGES MVA that is capable of producing geraniol. Based on the GC‐MS analysis, four G8Hs enabled increased production of 8‐hydrxygeraniol compared with CrG8H, and the superior two were TeG8H from *Tabernaemontana elegans* and SsG8H_2 from *Strychnos spinosa*, with 4.3‐ and 3.0‐fold increase compared with CrG8H (Figure 4C).

### The N‐terminus of TeG8H affects the G8H activity on 8‐dyroxygeraniol synthesis

To test whether the improved production of 8‐hydroxygeraniol catalyzed by TeG8H and SsG8H_2 was due to the good expression of these enzymes in the yeast cell, we constructed fusion proteins with six histidine residues (6× His tag) to measure the cellular abundance of CrG8H, TeG8H, and SsG8H_2 using Western blot (Figure S6A). To our surprise, we found that the protein levels of TeG8H and SsG8H_2 were significantly lower than that of CrG8H (Figure S6B). In addition, we found that yeast strains expressing CrG8H and TeG8H exhibited similar doubling time (Figure S7), indicating that the inefficient production of 8‐hydroxygeraniol by CrG8H was not related to the cell burden caused by its high expression in heterologous organisms.

**Figure 5:**
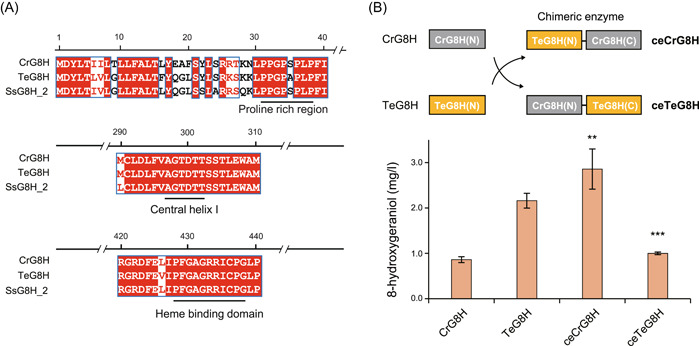
Analysis of the N‐terminal effect on geraniol 8‐hydroxylase (G8H) activity. (A) Multiple sequences alignment of CrG8H, TeG8H, and SsG8H_2. Proline‐rich region, central helix I, and heme‐binding domain are three functional domains of CrG8H. Amino acids with high conservation are indicated by red boxes. (B) The effect of the N‐terminal sequence of CrG8H and TeG8H proteins on 8‐hydroxygeraniol yield. The above panel shows the schematic diagram of N‐terminal amino acid exchange between CrG8H and TeG8H. The N‐terminal of CrG8H (CrG8H(N), 1–29 aa) and TeG8H (TeG8H(N), 1–29 aa) were ligated to the C‐terminal of the other G8H by over‐extension PCR reaction. The below panel shows the 8‐hydroxygeraniol production of strains before and after exchange. The 8‐hydroxygeraniol yield in fermentation culture of strains was determined using GC‐MS. Error bars show the mean ± standard error of three biological replicates. Student's two‐tailed *t*‐test was used for significance test (**p < 0.01, ***p < 0.001). The CrG8H was as control for significance test of ceCrG8H, and the TeG8H was as control for significance test of ceTeG8H.

To further elucidate this observation, we analyzed the primary sequence of these three enzymes. The functional domains of G8H enzymes were found to be quite conserved except for a few differences at their N‐terminal region (Figure 5A). As the transmembrane helical structure at the N‐terminal of G8H is known to play an important role in cellular localization of the endoplasmic reticulum (ER) in yeast^29^, ^30^, we hypothesized that the difference of the N‐terminal sequence in G8H enzymes might be responsible for their different performance in yeast cells. To test this idea, we swapped the N‐terminal sequence of CrG8H and TeG8H, resulting in two chimeric enzymes called ceCrG8H and ceTeG8H (Figure 5B). We found that utilization of ceCrG8H, a CrG8H variant that harbors the N‐terminus sequence from TeG8H, could produce 8‐hydroxygeraniol at an even higher level than that of the superior TeG8H. On the contrary, expressing ceTeG8H that consists of the N‐terminus of CrG8H and the remaining sequence from TeG8H in yeast cells could only synthesize a small amount of 8‐hydroxygeraniol at a similar level to that of CrG8H (Figure 5B). To study the effect of N‐terminal sequence on protein expression, we performed Western blot analysis to measure the cellular abundance of ceCrG8H and ceTeG8H enzymes and found that the expression level of ceCrG8H is much lower than ceTeG8H (Figure S6C). These findings indicate a negative correlation between the expression level of G8H enzymes and the in vivo performance, which is also consistent with our data using CrG8H and TeG8H. To further evaluate this intriguing observation, we adjusted the expression of CrG8H by using three well‐characterized promoters with varying promoter strength (pCYC1 < pTEF2 < pTDH3). Consistent with our hypothesis, we found that the level of 8‐hydroxygeraniol yield decreased in response to the increase in promoter strength (Figure S8). Overall, these results indicated that the N‐terminus of TeG8H leads to the increased activity of G8H for 8‐hydroxygeraniol biosynthesis in yeast cells.

### Construction of the final strain for efficient 8‐hydroxygeraniol production

**Figure 6:**
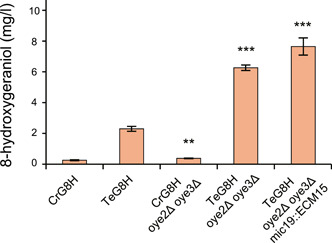
The comparison of 8‐hydroxygeraniol production of different strains. CrG8H: ypl062w::tCrGES MVA strain expressing CrG8H on episomal plasmid; TeG8H: ypl062w::tCrGES MVA strain expressing TeG8H on episomal plasmid; CrG8H oye2Δ oye3Δ: CrG8H stain with *OYE2* and *OYE3* genes knock‐out; TeG8H oye2Δ oye3Δ: TeG8H stain with *OYE2* and *OYE3* genes knock‐out; TeG8H oye2Δ oye3Δ mic19::ECM15: TeG8H oye2Δ oye3Δ strain with geraniol regulatory targets *MIC19* knock‐out and *ECM15* overexpressed. The 8‐hydroxygeraniol yield in fermentation culture of strains was determined using GC‐MS. Error bars show the mean ± standard error of three biological replicates. Student's two‐tailed *t*‐test was used for significance test (**p < 0.01, ***p < 0.001).

For further improvement of 8‐hydroxygeraniol yield, we combined different targets that promote geraniol biosynthesis based on this and previous studies. First, we tried to eliminate the competitive pathway of 8‐hydroxygeraniol synthesis in yeast by knocking out two endogenous genes encoding OYE2 and OYE3 enzymes that could convert geraniol to other compounds as mentioned previously^31^. We found that the 8‐hydroxygeraniol yield was increased by 1.5‐ fold in the *OYE2* and *OYE3* double mutant expressing CrG8H compared with CrG8H strain, and the 8‐hydroxygeraniol yield was increased by 2.7‐fold in the *OYE2* and *OYE3* double mutant expressing TeG8H compared with TeG8H strain (Figure 6). We then transplanted the high‐yield geraniol targets (*MIC19* deletion and *ECM15* duplication) identified by SCRaMbLE into the double mutant strain expressing TeG8H. These modifications resulted in a further slight improvement in 8‐hydroxygeraniol yield (Figure 6). The final stain (TeG8H oye2∆ oye3∆ mic19::ECM15) produced 7.65 mg/l 8‐hydroxygeraniol, which was 30.6‐fold higher than the production of the initial producing strain CrG8H (0.25 mg/l). These results demonstrated the effectiveness of our collaborative strategy to boost 8‐hydroxygeraniol biosynthesis pathway in yeast cells by both chassis engineering and key enzyme optimization.

## DISCUSSION

Our study achieved efficient production of 8‐hydroxygeraniol in budding yeast through different approaches, including optimization of MVA and geraniol biosynthetic pathways, modifications of newly discovered targets by SCRaMbLE, and utilization of superior G8Hs via genome mining. As 8‐hydroxygeraniol serves as the key precursor of MIAs, we envision that future studies aiming to increase the yield of high‐value natural products belonging to MIAs (e.g., vinblastine) would also benefit from the findings in this work.

Developing a high‐throughput screening method is essential to identify strains of interest from SCRaMbLEd pools with massive genome rearrangements. In this study, we established a facile method to identify post‐SCRaMbLE strain variants with improved yield of geraniol, a colorless metabolite, by coupling geraniol production and GeDH‐mediated NAD^+^ to NADH conversion in an in vitro reaction. The specificity of GeDH is essential for the high‐throughput screening method developed in this study. Since previous works have shown that the GeDH enzyme has very high specificity for geraniol and could distinguish ethanol^17^ or other metabolites with similar structures (citronellol/nerol)^32^, we envision that our method would be useful for other relevant studies. Although beneficial mutations were revealed to enhance the production of geraniol, the effect of these structural variations was not significant. We envision that utilization of the final Sc2.0 yeast strains harboring all synthetic chromosomes would further unleash the potential of SCRaMbLE to identify more beneficial targets. In addition, we think higher throughput screening methods and a great strain diversity would be useful to increase the likelihood of obtaining SCRaMbLEd strains with higher efficiency of geraniol biosynthesis.

Two discovered targets (*MIC19* deletion and *ECM15* duplication) were found to improve the production of geraniol. Although the effect of *MIC19* or *ECM15* on monoterpene synthesis was unclear based on previous literature, we found that the terpenoid backbone biosynthesis pathway is upregulated in SCRaMbLEd or genetically modified strains with *MIC19* deletion or/and *ECM15* duplication based on transcriptome analysis (Table S4). Notably, the expression of *YJL167W* (*ERG20*) gene, which encodes the key enzyme to catalyze GPP generation, was also increased. Thus, we speculated that enhanced geraniol production involving the two beneficial targets might be due to *ERG20* overexpressing.

The newly discovered TeG8H and SsG8H_2 proteins showed significantly higher activity than the commonly used CrG8H to produce 8‐hydroxygeraniol in yeast cells. Besides TeG8H and SsG8H_2, we also found two G8H homologs (AhG8H and CiG8H) with increased efficiency compared with CrG8H. These findings indicated that genomic information of native plants serves as a huge treasure trove to search superior enzymes involved in natural product biosynthesis. TeG8H, AhG8H, and CrG8H were all identified from *Apocynaceae*; plants in this family are known to produce various types of MIAs. For instance, at least 66 types of MIAs have been identified in *T. elegans* ^33^. SsG8H_2 was identified from *S. spinosa*, which is rich in strychnine, a type of MIA, accounting for 39.0% of the total alkaloids. We think genome mining of rate‐limiting enzymes would be a more effective and powerful strategy to optimize the biosynthesis pathway compared with the traditional way by increasing the copy number of low‐activity genes. Although different G8H enzymes showed distinct performance in yeast cells, we noticed high conservation of these enzymes except for the N‐terminus. By swapping the N‐terminal segments of representative G8H enzymes, we found that this region plays a key role in determining the cellular G8H activity on 8‐dyroxygeraniol synthesis in budding yeast, possibly by affecting protein expression. In addition, we hypothesized that differences in N‐terminal sequences of cytochrome P450 enzymes might affect their cellular localization and protein processing such as glycosylation, substrate conversion rate, or interaction with the coenzyme as suggested in previous studies^34^, ^35^.

Although we achieved significant improvement in 8‐hydroxygeraniol biosynthesis compared to the initial stain (~30‐fold), the ability to produce 8‐hydroxygeraniol by our final strain is still inferior to the engineered yeast strains reported in other studies (Table S1). One reason might be related to the optimization of G8H reaction conditions, like coenzyme CPR matching, cofactor NADPH increasing, and located organelle (ER) engineering, which would improve enzymatic efficiency as well. Besides, previous studies reviewed that *ERG8, ERG12, ERG13,* and *ERG19* genes on the MVA pathway could enhance geraniol supply to further increase 8‐hydroxygeraniol production^9^, ^10^, ^31^. Another potential improvement could be the optimization of fermentation conditions, and larger fermentation scale and carbon restriction strategy would benefit secondary metabolite productivity of strains. By combining aforementioned strategies and regulatory targets discovered in this study, we envision that the biosynthesis of 8‐hydroxygeraniol and MIAs in *S. cerevisiae* could be further improved.

## MATERIALS AND METHODS

### Strains and media

The *S. cerevisiae* strains used in this study are listed in Table S5. Wild‐type yeast strain BY4741 was used for geraniol or 8‐hydroxygeraniol production. Synthetic yeast strain syn2369R (*synII*, *synIII*, *synVI*, and *synIXR*) was used for SCRaMbLE evolution. Yeasts were cultured at 30°C in Yeast Extract Peptone Dextrose (YPD) medium (20 g/l peptone, 10 g/l yeast extract, and 20 g/l glucose) or SC medium (synthetic complete medium lacking appropriate amino acid with 20 g/l glucose) or supplemented with 500 mg/l G418 sulfate (GENVIEW, AG138‐100MG), 50 mg/l zeocin (Invitrogen, R25001), and 200 mg/l Hygromycin B (MCE, HY‐B0490) for KanMX, BleoR, and Hph marker screening, respectively.

*Escherichia coli* strain DH5α, used for plasmid construction and amplification, or strain BL21, used for protein expression, was cultured at 37°C in Luria–Bertani (LB) medium supplemented with 50 mg/l Carbenicillin (BBI, A600469‐0005) or 100 mg/l Kanamycin (BBI, A600286‐0025) for selection.

### Construction of plasmids and strains

The plasmids constructed or used in this study are listed in Table S6. The primers used for DNA amplification or Sanger sequencing in this study are listed in Table S7. DNA polymerase, restriction endonucleases, and T4 DNA ligase used for plasmid construction were purchased from New England Biolabs. The information for genes encoding heterologous enzymes (GESs, G8Hs, GeDH, and IDI) is listed in Table S8. The DNA sequence of GESs, G8Hs, and IDI were codon‐optimized for expression in *S. cerevisiae* and synthesized by GCATbio Co., Ltd. (Cat number: LS‐GS‐00001). Then they were ligated into HcKan_O and assembled with promoters and terminators into POTs or pRS series vectors using the YeastFab method^36^. Different promoters (pGAL1, pGAL7, and pGAL10) or terminator (tTEF2) was amplified from BY4741 genome and then ligated into HcKan_P or HcKan_T vector using the YeastFab method as well.

The yeast homologous recombination was used to engineer the yeast genome. For gene encoding GES integration at *YPL062W* locus, the 285 bp upstream and 320 bp downstream homologous sequence of *YPL062W* were amplified and added to *tCrGES* through PCR reaction. For MVA pathway gene integration at *the GAL80* locus, the three gene transcriptional units were digested by BsmBI from plasmid pRS415‐MVA and combined with LEU2 marker and the 500 bp upstream and 500 bp downstream homologous sequence of *GAL80*. For reconstructing genomic structural variation, the selection marker was integrated at the gene locus with 60 bp homologous arm of *LSB3* or *MIC19* to deletion or combined with inversion of *CEN6* sequence by overlap extension‐PCR. While *ECM15* was ligated to pRS413 or combined with the selection marker and homologous arm for duplication. For knocking out genes on competitive pathway, *tObGES* transcriptional unit was combined with KanMX or BleoR marker and 60 bp upstream or downstream of *OYE2* or *OYE3* gene, then transformed PCR product to yeast to modify genome.

### Geraniol‐producing synthetic strain SCRaMbLE

The pRS413‐Cre/EBD plasmid was transferred into the geraniol‐producing synthetic yeast strain syn2369R ypl062w::tCrGES MVA. As Cre recombinase was leaked without inducing, chromosomal variation occurs in the starting strain, which will be mistaken as a common variation of high‐yield strains. Multiple independent SCRaMbLE reactions were carried out to reduce the impact of starting strain variation. Positive transformant colonies were inoculated into 500 μl SC–Ura–Leu–His medium in a 96‐well deep well plate and incubated overnight at 30°C and 220 rpm. The precultures were transferred into 1 ml fresh medium supplemented with 1 μM 17‐β‐estradiol (E888000; Toronto Research Chemicals) with an initial OD_600_ of 0.1. The culture was shaken at 30°C and 220 rpm to induce SCRaMbLE for 24 h. After SCRaMbLE, about 200 colonies were plated on each SC–Ura–Leu agar plate and incubated at 30°C for 3 days.

### Screening of high‐yield geraniol strains

SCRaMbLEd strains were randomly selected and transferred into a 96‐well deep well plate containing 1 ml SC–Ura–Leu medium. After shaking at 30°C and 220 rpm for 24 h, they were transferred to 1 ml fresh medium with initial OD_600_ of 0.1 and shook at 30°C and 220 rpm for 48 h. The fermentation broth was centrifuged at 2000 rpm for 20 min to separate the supernatant and strain precipitation. The GeDH enzymatic condition was according to previous studies^17^. Fifty microliters supernatant was transferred and mixed with 2× GeDH enzyme reaction buffer (200 mM Tris–HCl (pH 8.0), 4 mM NAD^+^, 0.054 U GeDH) using automated liquid transfer workstation (Hamilton, Microlab STAR). The absorption at 340 nm was detected within 30 min using microplate reader (Synergy H1; BioTek), and the supernatant of each plate was detected three times.

### Geraniol and 8‐hydroxygeraniol fermentation and quantification

Wild‐type or synthetic yeast strains to be fermented were inoculated into 5 ml appropriate SC medium. Precultures were shaken overnight at 30°C and 220 rpm, then the seed culture was transferred into 10 ml fresh medium with initial OD_600_ of 0.1 and fermented at 30°C and 220 rpm for 5 days.

For the extraction of geraniol or 8‐hydroxygeraniol, the fermentation broth was centrifuged at 4000 rpm for 10 min, and then 700 μl of supernatant was collected and vortexed with 300 μl organic extractant (acetone:ethyl acetate = 1:3) for 2 min. The mixture was centrifuged at 12,000 rpm for 5 min, then the upper organic phase was collected and filtrated through a 0.22 μm organic filter membrane.

The extract samples were analyzed using GC‐MS to quantify geraniol and 8‐hydroxygeraniol production, equipped with HP‐FFAP chromatographic column (0.32 mm × 0.5 μm × 25 m; Agilent). Helium was used as the carrier gas, with a split ratio of 20:1 and a flow rate of 1.5 ml/min. The injection volume was 1 μl and the inlet temperature was 260°C. The column temperature was initially held at 60°C for 5 min, then ramped at 60°C/min to 150°C and 15°C/min to 220°C, then maintained for 10 min. The mass spectrometry ion source was EI+, the ion source and transmission line temperature was 290°C, and the solvent delay was 3.5 min. The SIM mode was used for collection, with geraniol in cationic mode and characteristic fragments of 41, 68, 69, and 123 *m*/*z* and 8‐hydroxygeraniol in cationic mode and characteristic fragments of 41, 43, 68, and 84 *m*/*z*.

### Genomic DNA extraction and structural variation analysis

A single colony of the SCRaMbLEd candidate strains was inoculated into 5 ml YPD medium at 30°C and 220 rpm for 48 h, and then the yeast genomic DNA was purified using the phenol‐chloroform extraction and isoamyl alcohol precipitation method. The whole genome was sequenced using PE100 double‐ended sequencing (DIPSEQ; BGI), with 1 Gb data of each strain (~85‐fold genome coverage).

For data process, low‐quality reads (Phred‐score < 10) or reads with unknown bases were removed using SOAPnuck v1.5.6 software, with less than 15% of reads filtered. Then the filtered data were mapped against reference sequences of synthetic chromosomes, *synII* ^37^, *synIII* ^38^, *synVI* ^39^, *synIXR* ^22^, and wild‐type chromosomes in BY4741 (Genbank No.: JRIS00000000) using SOAP aligner. The unmapped data with the loxPsym site (breakpoint) and containing at least 15 bp flanked were divided into two fragments from the loxPsym site. The spit reads were aligned with the reference sequence using Bowtie2 to locate the new collection point besides the loxPsym site. More than five breakpoint sequences were recorded as SCRaMbLE events, and then the SCRaMbLE event type was determined by breakpoint direction and sequence coverage depth.

### RNA extraction and transcriptome analysis

Yeasts to be RNA sequenced were grown in appropriate SC medium at 30°C and 220 rpm to an OD_600_ of 3–5, then RNA was extracted using the Yeast RNA kit (R6870‐02; OMEGA BioTek). The RNA sequencing was carried out by BGI, and the software SOAPnuke, HISAT2, SAMtools, and DESeq2 were used to process the RNA‐seq data. The log_2_ fold change greater than 0 was considered an upregulated gene, while log_2_ fold change less than 0 was considered a downregulated gene. The pathway enrichment was performed on differentially expressed genes in the Kyoto Encyclopedia of Genes and Genomes database.

### Growth measurement and doubling time analysis

Each yeast cell was inoculated in triplicate in a microplate with 200 μl of the appropriate SC medium, starting with an initial OD_600_ of 0.01. The strains were oscillated at 30°C and 200 rpm for 30 h. The kinetic growth of the strains was monitored using a Bioscreen C system (Lab Systems), with the OD_600_ measured at 20‐min intervals. The doubling times of the strains were calculated as previously described^40^.

### G8Hs protein screening

G8H homologous gene mining process is shown in Figure S5. Based on the sequence identity and other criteria, the process was carried out in 1000 plant transcriptome project (1 KP) and NCBI Nucleotide Sequence Database (NT). *CrG8H* was used as the query sequence, and the screening criteria included bit score >1000, percentage of identical matches >75, and alignment length >1000 to obtain a preliminary list of genes. Next, check if it was an Open Reading Frame (ORF) with protein‐encoding and extract the longest ORF. Finally, determine whether the protein was from the MIA‐producing plant, then output homologous sequences that meet the requirements.

### Multiple protein sequence alignment

Multiple homologous sequence alignment of GES or G8H from different sources was performed using ClustalW, and the alignment results were output using ESPript (http://espript.ibcp.fr/ESPript/cgi‐bin/ESPript.cgi). The phylogenetic tree was analyzed using Neighbor‐Joining tree on MEGA‐11 software.

### GeDH purifying and characterization

Geraniol dehydrogenase from *Castellaniella defragrans* was synthesized and ligated into pET28a vector by BGI. Transfer the plasmid into *E. coli* BL21 (DE3) cells, then induce protein expression by 0.1 mM IPTG for 20 h at 16°C. The protein was purified using nickel ion affinity chromatography (ÄKTA pure; GE Healthcare) after cell lysis and then detected using sodium dodecyl sulfate polyacrylamide gel electrophoresis (SDS‐PAGE) and Western blot.

To character purified GeDH enzyme activity, different concentrations of geraniol (0, 0.0625, 0.125. 0.25, 0.5, and 1 mM) were prepared in culture medium or fermentation broth as substrate, then mixed with 50 μl GeDH reaction solution (100 mM Tris–HCl [pH 8.0], 2 mM NAD^+^, 0.181 µg GeDH protein). The OD_340_ was continuously measured for 30 min, with reading every 30 s and oscillating for 10 s before measurement. Finally, create an enzyme reaction curve and calculate the GeDH‐specific enzyme activity.

### SDS‐PAGE and Western blot analysis

Ten OD_600_ of cell pellets were mixed with 200 µl of 0.1 M NaOH and stayed at room temperature for 5 min and then centrifuged at 12,000 rpm for 1 min. The precipitate was resuspended in 100 µl of 2× SDS‐PAGE loading buffer, boiled for 10 min, and then centrifuged at 12,000 rpm for 3 min. Five microliters of supernatant was separated on 10% SDS‐PAGE gel followed by transferred onto polyvinylidene difluoride membranes for Western blot. HRP anti‐6× His tag antibody (Abcam or ABclonal) was used to detect expression of his‐tagged G8H proteins.

## AUTHOR CONTRIBUTIONS

**Yu Zhang**: Conceptualization (equal); data curation (lead); funding acquisition (equal); investigation (lead); project administration (equal); validation (lead); visualization (equal); writing—original draft (lead). **Mengdi Yuan**: Data curation (equal); investigation (equal); validation (equal). **Xinxin Wu**: Data curation (equal); investigation (equal); validation (equal). **Qiuhui Zhang**: Investigation (equal). **Yuzhu Wang**: Investigation (equal). **Liming Zheng**: Investigation (equal). **Tsan‐Yu Chiu**: Project administration (equal); supervision (equal). **Huiming Zhang**: Formal analysis (lead); software (equal). **Lei Lan**: Software (lead). **Feng Wang**: Data curation (equal); investigation (equal); validation (supporting). **Ying Liao**: Investigation (equal); validation (supporting). **Xuemei Gong**: Investigation (supporting). **Shirui Yan**: Investigation (supporting). **Yun Wang**: Visualization (equal). **Yue Shen**: Funding acquisition (equal); project administration (equal); resources (lead); supervision (lead); writing—review and editing (equal). **Xian Fu**: Conceptualization (equal); project administration (equal); resources (lead); supervision (lead); writing—original draft (equal); writing—review and editing (lead).

## ETHICS STATEMENT

No animals or humans were involved in this study.

## CONFLICT OF INTERESTS

The authors declare the following competing interests: Y. Z., X. F., H. Z., T. C., Y. L., Y. S., and BGI Research have filed a patent application (Chinese patent application number: 202310783474.7) describing the new geraniol regulatory targets identified by SCRaMbLE in this study. The remaining authors declare no competing interests.

## Supporting information

Supporting information.

## Data Availability

The DNA sequencing data of this study is openly available in CNSA (CNGB Nucleotide Sequence Archive) under accession number CNP0004507.
