# Supplementary material for: The construction and optimization of engineered yeast chassis for efficient biosynthesis of 8‐hydroxygeraniol
Source: mLife. 2023 Dec 26;2(4):438–49. doi: 10.1002/mlf2.12099 (PMC10989129; doi:10.1002/mlf2.12099)
Supplement: Supplementary file 1 — Supporting information. [file MLF2-2-438-s001.docx]

**Supplementary Tables**

**Supplementary Table 1. Previous studies on the synthesis of** **8-hydroxygeraniol in yeast**

| **8-hydroxygeraniol titer** | **Fermentation condition** | **Reference** |
| --- | --- | --- |
| 5.3 mg/l | Flask | ^1^ |
| 52 mg/l | Culture tube | ^2^ |
| 227 mg/l | 2.0 l bioreactor | ^2^ |
| 25.11 mg/l | Flask | ^3^ |
| 92.8 mg/l/OD | 96-well deep well plate | ^4^ |
| 238.9 mg/l | Flask | ^5^ |
| 1 g/l | 5.0 l bioreactor | ^5^ |

**Supplementary Table 2. Structural variations observed in synthetic chromosomes of geraniol high-yield SCRaMbLEd strains**

| **strain** | **SV_Type** | **syn_chro** | **start** | **end** | **Genes involved** |
| --- | --- | --- | --- | --- | --- |
| SCR467 | del | *synII* | 432057 | 432375 | - |
| SCR850 | inv | *synII* | 50549 | 63576 | *YBL084C, YBL083C, YBL082C, YBL080C, YBL079W* |
|  | del | *synIII* | 209836 | 210025 | *YCR066W* |
| SCR860 | inv | *synVI* | 135161 | 138344 | *YFR007W, YFR008W* |
|  | inv | *synIXR* | 329990 | 335092 | *YIR006C* |
| SCR1789 | del | *synVI* | 177691 | 179666 | *YFR024C-A* |
| SCR1813 | del | *synIXR* | 73421 | 73640 | *YCL024W 3’UTR* |
| SCR1900 | del | *synVI* | 142692 | 143594 | *YFR010W-A, YFR011C* |
|  | del | *synIXR* | 389154 | 389371 | *YIR037W 3‘UTR, YIR038C 3’UTR* |
| SCR1987 | del | *synIII* | 45669 | 45755 | - |
|  | del | *synVI* | 135178 | 138363 | *YFR007W, YFR008W* |
|  | del | *synIII* | 45635 | 45721 | - |
| SCR1995 | dup | *synII* | 214742 | 215635 | *YBL001C* |
| SCR1997 | inv | *synVI* | 125614 | 125843 | CEN6 |
| SCR2029 | inv | *synVI* | 125606 | 125851 | CEN6 |
|  | del | *synII* | 720948 | 721156 | - |
| SCR2031 | del | *synIII* | 100531 | 100962 | *YCR003W 3‘UTR* |
|  | del | *synII* | 627712 | 628054 | - |
|  | inv | *synVI* | 125610 | 125845 | CEN6 |

Note: “del” means deletion, “dup” means duplication, “inv” means inversion, “-” means no genes involvement.

**Supplementary Table 3. 18 kinds of G8H genes from MIAs producing medicinal plants**

| **G8H** | **Sequence identity** | **Source plant** | **Plant family** |
| --- | --- | --- | --- |
| *TeG8H* | 81.714% | *Tabernaemontana elegans* | *Apocynaceae* |
| *AhG8H* | 81.522% | *Amsonia hubrichtii* | *Apocynaceae* |
| *RsG8H* | 81.377% | *Rauvolfia serpentina* | *Apocynaceae* |
| *VmG8H* | 80.769% | *Vinca minor* | *Apocynaceae* |
| *WnG8H* | 78.983% | *Wrightia natalensis* | *Apocynaceae* |
| *GsG8H* | 79.133% | *Gelsemium sempervirens* | *Gelsemiaceae* |
| *SsG8H_1* | 78.668% | *Strychnos spinosa* | *Loganiaceae* |
| *SsG8H_2* | 78.378% | *Strychnos spinosa* | *Loganiaceae* |
| *HpG8H* | 79.5% | *Holarrhena pubescens* | *Apocynaceae* |
| *OpG8H* | 77.702% | *Ophiorrhiza pumila* | *Rubiaceae* |
| *CcG8H* | 77.365% | *Cinchona calisaya* | *Rubiaceae* |
| *SmG8H* | 77.913% | *Swertia mussotii* | *Gentianceae* |
| *CeG8H* | 77.228% | *Coffea eugenioides* | *Rubiaceae* |
| *CaG8H* | 77.086% | *Coffea arabica* | *Rubiaceae* |
| *GaG8H* | 76.662% | *Gentiana acaulis* | *Gentianceae* |
| *CiG8H* | 75.563% | *Carapichea ipecacuanha* | *Rubiaceae* |
| *SaG8H* | 76.504% | *Swertia asarifolia* | *Gentianceae* |
| *EaG8H* | 76.194% | *Exacum affine* | *Gentianceae* |

**Supplementary Table 4. The RNA-seq analysis of differentially expressed genes on terpenoid backbone biosynthesis pathway in geraniol high-yield strains**

| **Stains** | **log2FoldChange** | | | | | | | | | | **Upregulated Genes Count** | **Downregulated Genes Count** | **Total Genes Count** | **Up/Down-Regulation** |
| --- | --- | --- | --- | --- | --- | --- | --- | --- | --- | --- | --- | --- | --- | --- |
|  | **YPL028W** | **YLR450W** | **YMR208W** | **YMR220W** | **YNR043W** | **YJL167W** | **YBR003W** | **YDL193W** | **YKL019W** | **YJR117W** |  |  |  |  |
| SCR1900 | -0.05736 | 0.582978 | -0.14656 | 0.033037 | -0.14588 | 1.674481 | 0.216053 | 0.248711 | 0.108287 | 0.05677 | 7 | 3 | 10 | Up |
| SCR1995 | 0.113885 | 0.559551 | -0.40554 | 0.000813 | -0.26379 | 1.975816 | 0.185675 | 0.267294 | 0.109281 | -0.0757 | 7 | 3 | 10 | Up |
| delMIC19 | -0.03693 | 0.259035 | -0.03327 | -0.30501 | 0.047615 | 1.510876 | -0.14747 | 0.447507 | 0.280821 | -0.01071 | 5 | 5 | 10 | Up |
| dupECM15 | -0.17163 | 0.100574 | 0.175641 | -0.07727 | 0.290692 | 1.543378 | 0.130655 | 0.221625 | -0.012 | 0.100471 | 7 | 3 | 10 | Up |
| mic19::ECM15 | -0.09893 | 0.079479 | 0.038079 | -0.17196 | 0.101122 | 1.484365 | -0.25754 | 0.29216 | 0.176087 | -0.15621 | 6 | 4 | 10 | Up |

**Supplementary Table 5. The yeast strains used in this study**

| **Stains** | **Genotype** | **Source** |
| --- | --- | --- |
| BY4741 | *MATa his3Δ1 leu2Δ0 LYS2 met15Δ0 ura3Δ0* | ^6^ |
| POT1 | *BY4741, POT1* | This study |
| tVoGES | *BY4741, POT1-tVoGES* | This study |
| tCrGES | *BY4741, POT1-tCrGES* | This study |
| tObGES | *BY4741, POT1-tObGES* | This study |
| ypl062w::tCrGES | *BY4741, ypl062w∆::pTDH3-tCrGES-tADH1, URA3* | This study |
| ypl062w::tCrGES MVA | ypl062w::tCrGES*, gal80∆:: pGAL1-ERG20ww-tCIT1， pGAL7- IDI-tFUM1，pGAL10- tHMG1-tERP2，LEU2* | This study |
| pRS413 | ypl062w::tCrGES MVA*, pRS413* | This study |
| CrG8H | ypl062w::tCrGES MVA*, pRS413-CrG8H* | This study |
| syn2369R | *MATα his3Δ1 leu2Δ0 ura3Δ0 synLYS2, MET15, HO::SUP61 synII, synIII, synVI, synIXR* | This study |
| syn2369R ypl062w::tCrGES MVA | *Syn2369R, ypl062w∆::pTDH3-tCrGES-tADH1, URA3, gal80∆:: pGAL1- ERG20ww-tCIT1， pGAL7- IDI-tFUM1，pGAL10- tHMG1-tERP2，LEU2* | This study |
| delLSB3 | syn2369R ypl062w::tCrGES MVA*, lsb3∆, HIS3* | This study |
| delMIC19 | syn2369R ypl062w::tCrGES MVA*, mic19∆, HIS3* | This study |
| dupECM15 | syn2369R ypl062w::tCrGES MVA*, pRS413-ECM15* | This study |
| invCEN6 | syn2369R ypl062w::tCrGES MVA*, cen6∆::invCEN6，HIS3* | This study |
| mic19::ECM15 | syn2369R ypl062w::tCrGES MVA*, mic19∆::ECM15，KanMX* | This study |
| mic19::ECM15 invCEN6 | *mic19::ECM15, cen6∆::invCEN6，HIS3* | This study |
| mic19::ECM15 delLSB3 | *mic19::ECM15, lsb3∆::BleoR* | This study |
| mic19::ECM15 invCEN6 delLSB3 | *mic19::ECM15, ∆cen6::invCEN6，HIS3, ∆lsb3::BleoR* | This study |
| TeG8H | ypl062w::tCrGES MVA*, pRS413-TeG8H* | This study |
| AhG8H | ypl062w::tCrGES MVA*, pRS413-AhG8H* | This study |
| RsG8H | ypl062w::tCrGES MVA*, pRS413-RsG8H* | This study |
| VmG8H | ypl062w::tCrGES MVA*, pRS413-VmG8H* | This study |
| WnG8H | ypl062w::tCrGES MVA*, pRS413-WnG8H* | This study |
| GsG8H | ypl062w::tCrGES MVA*, pRS413-GsG8H* | This study |
| CrG8H | ypl062w::tCrGES MVA*, pRS413-CrG8H* | This study |
| SsG8H-1 | ypl062w::tCrGES MVA*, pRS413-SsG8H-1* | This study |
| SsG8H-2 | ypl062w::tCrGES MVA*, pRS413-SsG8H-2* | This study |
| HpG8H | ypl062w::tCrGES MVA*, pRS413-HpG8H* | This study |
| OpG8H | ypl062w::tCrGES MVA*, pRS413-OpG8H* | This study |
| CcG8H | ypl062w::tCrGES MVA*, pRS413-CcG8H* | This study |
| SmG8H | ypl062w::tCrGES MVA*, pRS413-SmG8H* | This study |
| CeG8H | ypl062w::tCrGES MVA*, pRS413-CeG8H* | This study |
| CaG8H | ypl062w::tCrGES MVA*, pRS413-CaG8H* | This study |
| GaG8H | ypl062w::tCrGES MVA*, pRS413-GaG8H* | This study |
| CiG8H | ypl062w::tCrGES MVA*, pRS413-CiG8H* | This study |
| SaG8H | ypl062w::tCrGES MVA*, pRS413-SaG8H* | This study |
| EaG8H | ypl062w::tCrGES MVA*, pRS413-EaG8H* | This study |
| CrG8H-His6 | ypl062w::tCrGES MVA*, pRS413-CrG8H-His6* | This study |
| TeG8H-His6 | ypl062w::tCrGES MVA *, pRS413-TeG8H-His6* | This study |
| SsG8H_2-His6 | ypl062w::tCrGES MVA*, pRS413-SsG8H_2-His6* | This study |
| ceCrG8H | ypl062w::tCrGES MVA*, pRS413-TeG8H(N)-CrG8H(C)* | This study |
| ceTeG8H | ypl062w::tCrGES MVA*, pRS413-CrG8H(N)-TeG8H(C)* | This study |
| ceCrG8H(C)_2 | ypl062w::tCrGES MVA*, pRS413-SsG8H_2(N)-CrG8H(C)* | This study |
| ceSsG8H_2 | ypl062w::tCrGES MVA*, pRS413-CrG8H(N)-SsG8H_2(C)* | This study |
| pCYC1-CrG8H | ypl062w::tCrGES MVA*, pRS413-pCYC1-CrG8H* | This study |
| pTEF2-CrG8H | ypl062w::tCrGES MVA*, pRS413-pTEF2-CrG8H* | This study |
| pTDH3-CrG8H | ypl062w::tCrGES MVA*, pRS413-pTDH3-CrG8H* | This study |
| CrG8H oye2Δ oye3Δ | ypl062w::tCrGES MVA, *∆oye2:: pTDH3- tObGES-tADH1，KanMX*, *∆oye3:: pCCW12- tObGES-tTEF2，BleoR, pRS413-CrG8H* | This study |
| TeG8H oye2Δ oye3Δ | ypl062w::tCrGES MVA, *∆oye2:: pTDH3- tObGES-tADH1，KanMX*, *∆oye3:: pCCW12- tObGES-tTEF2，BleoR,*, *pRS413-TeG8H* | This study |
| TeG8H oye2Δ oye3Δ mic19::ECM15 | TeG8H oye2Δ oye3Δ, *mic19∆::ECM15，KanMX* | This study |

**Supplementary Table 6. The plasmids used in this study**

| **Plasmids** | **Genotype** | **Source** |
| --- | --- | --- |
| HcKan_O | ORF receiving vector | ^7^ |
| HcKan_P | promoter receiving vector | ^7^ |
| HcKan_T | terminator receiving vector | ^7^ |
| POT1 | transcription unit receiving vector | ^7^ |
| POT2 | transcription unit receiving vector | ^7^ |
| POT4 | transcription unit receiving vector | ^7^ |
| POT5 | transcription unit receiving vector | ^7^ |
| pRS413 | metabolic pathway receiving vector | ^8^ |
| pRS415 | metabolic pathway receiving vector | ^8^ |
| HcKan_P-pTDH3 | HcKan_P carrying pTDH3 promoter | ^7^ |
| HcKan_P-pGAL1 | HcKan_P carrying pGAL1 promoter | This study |
| HcKan_P-pGAL7 | HcKan_P carrying pGAL7 promoter | This study |
| HcKan_P-pGAL10 | HcKan_P carrying pGAL10 promoter | This study |
| HcKan_P-pCCW12 | HcKan_P carrying pCCW12 promoter | ^9^ |
| HcKan_P-pCYC1 | HcKan_P carrying pCYC1 promoter | ^7^ |
| HcKan_P-pTEF2 | HcKan_P carrying pTEF2 promoter | ^7^ |
| HcKan_T-tADH1 | HcKan_T carrying tADH1 terminator | ^7^ |
| HcKan_T-tCIT1 | HcKan_T carrying tCIT1 terminator | ^9^ |
| HcKan_T-tFUM1 | HcKan_T carrying tFUM1 terminator | ^9^ |
| HcKan_T-tERP2 | HcKan_T carrying tERP2 terminator | ^9^ |
| HcKan_T-tTEF2 | HcKan_T carrying tTEF2 terminator | This study |
| POT1-tVoGES | POT1-pTDH3-tVoGES-tADH1 | This study |
| POT1-tCrGES | POT1-pTDH3-tCrGES-tADH1 | This study |
| POT1-tObGES | POT1-pTDH3-tObGES-tADH1 | This study |
| pRS413-CrG8H | pRS413-pGAL1-CrG8H-tADH1 | This study |
| pRS415-MVA | pRS415-pGAL1-ERG20ww-tCIT1-pGAL7-IDI-tFUM1-pGAL10-tHMG1-tERP2 | This study |
| pRS413-CreEBD | pRS413 carrying Cre recombinase expression cassette fused with an estradiol-binding domain (EBD) | ^10^ |
| pRS413-ECM15 | pRS413-ECM15 transcriptional unit | This study |
| pRS413-TeG8H | pRS413-pGAL1-TeG8H-tADH1 | This study |
| pRS413-AhG8H | pRS413-pGAL1-AhG8H-tADH1 | This study |
| pRS413-RsG8H | pRS413-pGAL1-RsG8H-tADH1 | This study |
| pRS413-VmG8H | pRS413-pGAL1-VmG8H-tADH1 | This study |
| pRS413-WnG8H | pRS413-pGAL1-WnG8H-tADH1 | This study |
| pRS413-GsG8H | pRS413-pGAL1-GsG8H-tADH1 | This study |
| pRS413-SsG8H-1 | pRS413-pGAL1-SsG8H_1-tADH1 | This study |
| pRS413-SsG8H-2 | pRS413-pGAL1-SsG8H_2-tADH1 | This study |
| pRS413-HpG8H | pRS413-pGAL1-HpG8H-tADH1 | This study |
| pRS413-OpG8H | pRS413-pGAL1-OpG8H-tADH1 | This study |
| pRS413-CcG8H | pRS413-pGAL1-CcG8H-tADH1 | This study |
| pRS413-SmG8H | pRS413-pGAL1-SmG8H-tADH1 | This study |
| pRS413-CeG8H | pRS413-pGAL1-CeG8H-tADH1 | This study |
| pRS413-CaG8H | pRS413-pGAL1-CaG8H-tADH1 | This study |
| pRS413-GaG8H | pRS413-pGAL1-GaG8H-tADH1 | This study |
| pRS413-CiG8H | pRS413-pGAL1-CiG8H-tADH1 | This study |
| pRS413-SaG8H | pRS413-pGAL1-SaG8H-tADH1 | This study |
| pRS413-EaG8H | pRS413-pGAL1-EaG8H-tADH1 | This study |
| pRS413-CrG8H-His6 | pRS413-pGAL1-CrG8H fused with SSGHHHHHH amino acids-tADH1 | This study |
| pRS413-TeG8H-His6 | pRS413-pGAL1-TeG8H fused with SSGHHHHHH amino acids-tADH1 | This study |
| pRS413-SsG8H_2-His6 | pRS413-pGAL1-SsG8H_2 fused with SSGHHHHHH amino acids-tADH1 | This study |
| pRS413-ceCrG8H | pRS413-pGAL1-chimeric G8H with N-terminal of TeG8H and C-terminal of CrG8H-tADH1 | This study |
| pRS413-ceTeG8H | pRS413-pGAL1-chimeric G8H with N-terminal of CrG8H and C-terminal of TeG8H-tADH1 | This study |
| pRS413-pCYC1-CrG8H | pRS413-pCYC1-CrG8H-tADH1 | This study |
| pRS413-pTEF2-CrG8H | pRS413-pTEF2-CrG8H-tADH1 | This study |
| pRS413-pTDH3-CrG8H | pRS413-pTDH3-CrG8H-tADH1 | This study |

**Supplementary Table 7. The primers used in this study**

| **Primers** | **Sequence (5’-3’)** |
| --- | --- |
| tVoGES-F | AGCGTGCGTCTCAGATGCGTATCATGGAGCTTGTAGAGGC |
| tVoGES-R | GTGCTGCGTCTCGGCTATACTGAAACGCTGACTGGTGTG |
| pGAL1-F | AGCGTGGGTCTCAGGCTACGGATTAGAAGCCGCCGAGCGG |
| pGAL1-R | GTGCTGGGTCTCGCATCGGTTTTTTCTCCTTGACGTTAAA |
| tObGES-F | AGCGTGCGTCTCAGATGCGTAGGGAATACTTGCTAGAGGAGAC |
| tObGES-R | GTGCTGCGTCTCGGCTATTGTGTGAAGAACAGAGCATCGAC |
| tCrGES-F | AGCGTGCGTCTCAGATGAGGCGTGAATTGCTAGAAAGGAC |
| tCrGES-R | GTGCTGCGTCTCGGCTAGAAGCAAGGTGTAAAAAAAAGGGCC |
| ypl062w-HoL-F | CTCATATTCCTTCACTAGC |
| ypl062w-HoL-R | GCCCTTACGTGAGGGGCAGTG |
| ypl062w-HoL-URA3-F | CTGCCCCTCACGTAAGGGCTTCAATTCATCATTTTTTTTTTATTC |
| URA3-DOWN-R | TTGATAATGATAATAACTGATATAATTAAATTGAAGCTC |
| TDH3p-UP-F | TCAGTTATTATCATTATCAATACTGCCATTTCAAAG |
| TDH3p-DOWN-R | CATTATATTTAGTGGATGCCAGGAAT |
| ADH1t-UP-F | TAGCCGAATTTCTTATGATTTATGATT |
| tADH1-ypl062w-R | GAATTTGCCCACATGGTCGGTGCCGGTAGAGGTGTGGTCAATAAG |
| ypl062w-HoR-F | CACCGACCATGTGGGCAAATTC |
| ypl062w-HoR-R | GGTTCAGCAGTGTCAAAGTG |
| pGAL7-F | AGCGTGGGTCTCAGGCTGACGGTAGCAACAAGAATATAGC |
| pGAL7-R | GTGCTGGGTCTCGCATCTTTTGAGGGAATATTCAACTGTT |
| pGAL10-F | AGCGTGGGTCTCAGGCTTTATATTGAATTTTCAAAAATTC |
| pGAL10-R | GTGCTGGGTCTCGCATCAGTGGTTATGCAGCTTTTCCATT |
| Gal80up-F | ATGGACTACAACAAGAGATCTTCGG |
| Gal80up-Leu-R | TCCCACAGTTTGAGATATTAATTCTTTTGCTCTC |
| Leu-Gal80-F | TAATATCTCAAACTGTGGGAATACTCAGGTATCG |
| Leu-Gal1p-R | GCTTCTAATCCGTAGCCAGGTAGAGAAGGCCGTTTCTGACAGAGT |
| Gal80dn- ERP2t-F | CATCACTACATTCCTCTGAGACCTATGGCAATGTTCCAGTGTCATGC |
| Gal80dn-R | TTATAAACTATAATGCGAGATATTGCT |
| lsb3-HIS-F | GCAATTCGTTAATGATCCCTTTTGGGCCAATATATAATCTATAACTTCGTATAATGTACATCTATTACTCTTGGCCTCCTCTAGTAC |
| lsb3-HIS-R | CAAATCGAAGAAGCCGCTTCTAGCTTGGGTTTCAAAAGATGAATGATAACTTCGTATAATTGATGCATTACCTTGTCATCTTCAGT |
| mic19-HIS-F | TGATGGTTTATTACTTATATTTAAATATTGAAAGCTGCAAGATTTAAAAAAATAATAACTTCTATTACTCTTGGCCTCCTCTAGTAC |
| mic19-HIS-R | GATCTTAATGTATAAAGGATTTGGTCTGTAAGTCATAACTTCGTATAATGTACATTATACTGATGCATTACCTTGTCATCTTCAGT |
| ECM15 dup-F | GCAGTGACTCGGTCTCTACCTGGCTGAAGTTATGATTGATTGATCCTCTTGG |
| ECM15 dup-R | CAGTGTCTTGGGTCTCTCTCAGAGGCGTATAATGTACATTATACGAAGTTATCATTCAT |
| OE-HIS-F | ATACTCCAAGAAAAAGAACAACAAAAATAAGTAAAATATAACTTCGTATAATGTACATTATCTATTACTCTTGGCCTCCT |
| OE-HIS-R | GCAAAAAAAATAACTTCGTATTGATGCATTACCTTGTCATC |
| OE-HIS-invCEN6-F | ACAAGGTAATGCATCAATACGAAGTTATTTTTTTTGCTATTATAATACTAATTTC |
| OE-invCEN6-R | ACGTGTGTAGAGCCTTAAATTCTAATTTCTAACTTTAAATAACTTCGTATAATGTACATTTACGAAGTTATAATATATAAACCTGTATAATATAACCT |
| mic19-dupECM15-F | TGATGGTTTATTACTTATATTTAAATATTGAAAGCTGCAAGATTTAAAAAAATAATAACTGAAGTTATGATTGATTGATCCTCTTGG |
| OE-dupECM15-KanMX-R | GCCTCCATGTCCGTATAATGTACATTATACGAAGTTATCATTC |
| OE-KanMX-dupECM15-F | TACATTATACGGACATGGAGGCCCAGAATAC |
| mic19-KanMX-R | GATCTTAATGTATAAAGGATTTGGTCTGTAAGTCATAACTTCGTATAATGTACATTATACCAGTATAGCGACCAGCATTC |
| lsb3-BleoR-F | GCAATTCGTTAATGATCCCTTTTGGGCCAATATATAATCTATAACTTCGTATAATGTACAGACATGGAGGCCCAGAATAC |
| lsb3-BleoR-R | CAAATCGAAGAAGCCGCTTCTAGCTTGGGTTTCAAAAGATGAATGATAACTTCGTATAATCAGTATAGCGACCAGCATTC |
| G8H-histag-F | TCCTCCGGTCATCATCATCATCATCATTAGCCGAATTTCTTATGATTTATGAT |
| CrG8H-histag-R | ATGATGATGATGATGATGACCGGAGGAAAGGGTGCTTGGTACAG |
| TeG8H-histag-R | ATGATGATGATGATGATGACCGGAGGACAGCGGACTCGGTAC |
| SsG8H_2-histag-R | ATGATGATGATGATGATGACCGGAGGACAAAGGGATAGGTACGGC |
| Gi-GAL1p-F | CTTTAACGTCAAGGAGAAAAAACCG |
| Gi-CrG8H(N)-R | GCAAGGGGGCTGGGCCCGGTGGAAGATTTTTTGTACGACGACTTAGGTAACT |
| Gi-tTeG8H-F | CTTCCACCGGGCCCAG |
| Gi-GAL1p-R | CGGTTTTTTCTCCTTGACGTTAAAG |
| Gi-TeG8H(N)-R | GCAGGGGTGATGGGCCCGGCGGTAGTTTCTTAGACTTTCTGCTAAGGTAACTT |
| Gi-tCrG8H-F | CTACCGCCGGGCC |
| OE-OYE2int-F | GAGGTAGCAGATTCCTGGAACG |
| OE-OYE2int-(KanMX)-R | GGGCCTCCATGTCTATCGATACTATATTATCGTCTATATTTAGCTTAATATGATGATT |
| OE-KanMX-(OYE2int)-F | ATAGTATCGATAGACATGGAGGCCCAGAATACC |
| OE-KanMX-R | CGTATTCTTTGAAATGGCAGTATTGATAATGAAGCCAGGTCAGTATAGCGACCAGCATTCACAT |
| OE-OYE2int-(ADH1t)-F | GAGGTCGCTCTTATTGACCACACCTCTACCGGCCTCAGGCTAGTGTTAACCGTACTTTGTAGCACC |
| OE-OYE2int-R | TCTAAAAGAAGTTTATGTGAATAATGATCTAAAGTGAAAG |
| OE-OYE3int-F | TTGCGTTCCTGATATAACTCCGTTC |
| OE-OYE3-(BleoR)-R | GGGCCTCCATGTCCTTCTAAATTTAAACTTCGCTATACTGAACTACAG |
| OE-BleoR-(OYE3)-F | AATTTAGAAGGACATGGAGGCCCAGAATACC |
| OE-BleoR-(CCW12p)-R | GGATTCCCAAAACGGAAATCAGACGCCAATAGAGCCAGGTCAGTATAGCGACCAGCATTCACAT |
| OE-OYE3-(TEF2t)-F | TTTCTACTAACGTTTTCATTATTCTATACTCTCCTCAGGCAATCATGAATAAAGACATTGATGTTATGTATTTTTCATATTCC |
| OE-OYE3int-R | GCCCTATATAAACAAAGATCGAGTCTTTTTG |

**Supplementary Table 8. List of heterologous genes in this study**

| **Genes** | **Origin** | **Short Name** | **GenBank or reference^11^** |
| --- | --- | --- | --- |
| *GES* | *Valeriana officinalis* | VoGES | AHE41084.1 |
| *GES* | *Ocimum basilicum* | ObGES | AAR11765.1 |
| *GES* | *Catharanthus roseus* | CrGES | AFD64744.1 |
| *G8H* | *Catharanthus roseus* | CrG8H | CAC80883.1 |
| *G8H* | *Tabernaemontana elegans* | TeG8H | KF415107.1 |
| *G8H* | *Amsonia hubrichtii* | AhG8H | KF415105.1 |
| *G8H* | *Rauvolfia serpentina* | RsG8H | KF415106.1 |
| *G8H* | *Vinca minor* | VmG8H | KF415108.1 |
| *G8H* | *Wrightia natalensis* | WnG8H | gnl_onekp_EDEQ_scaffold_2013504 |
| *G8H* | *Gelsemium sempervirens* | GsG8H | gnl_onekp_HGSM_scaffold_2001976 |
| *G8H* | *Strychnos spinosa* | SsG8H_1 | gnl_onekp_GGJD_scaffold_2013247 |
| *G8H* | *Strychnos spinosa* | SsG8H_2 | gnl_onekp_GGJD_scaffold_2013245 |
| *G8H* | *Holarrhena pubescens* | HpG8H | gnl_onekp_JGYZ_scaffold_2045450 |
| *G8H* | *Ophiorrhiza pumila* | OpG8H | LC010422.1 |
| *G8H* | *Cinchona calisaya* | CcG8H | KF415104.1 |
| *G8H* | *Swertia mussotii* | SmG8H | GU168041.1 |
| *G8H* | *Coffea eugenioides* | CeG8H | XM_027298752.1 |
| *G8H* | *Coffea arabica* | CaG8H | XM_027222384.1 |
| *G8H* | *Gentiana acaulis* | GaG8H | gnl_onekp_ECTD_scaffold_2111925 |
| *G8H* | *Carapichea ipecacuanha* | CiG8H | gnl_onekp_BQEQ_scaffold_2009402 |
| *G8H* | *Swertia asarifolia* | SaG8H | KR709239.1 |
| *G8H* | *Exacum affine* | EaG8H | gnl_onekp_KPUM_scaffold_2102148 |
| *GeDH* | *Castellaniella defragrans* | CdGeDH | CCF55024.1 |
| *IDI* | *E.coli* | EcIDI | AF119715.1 |

**Supplementary Figures**


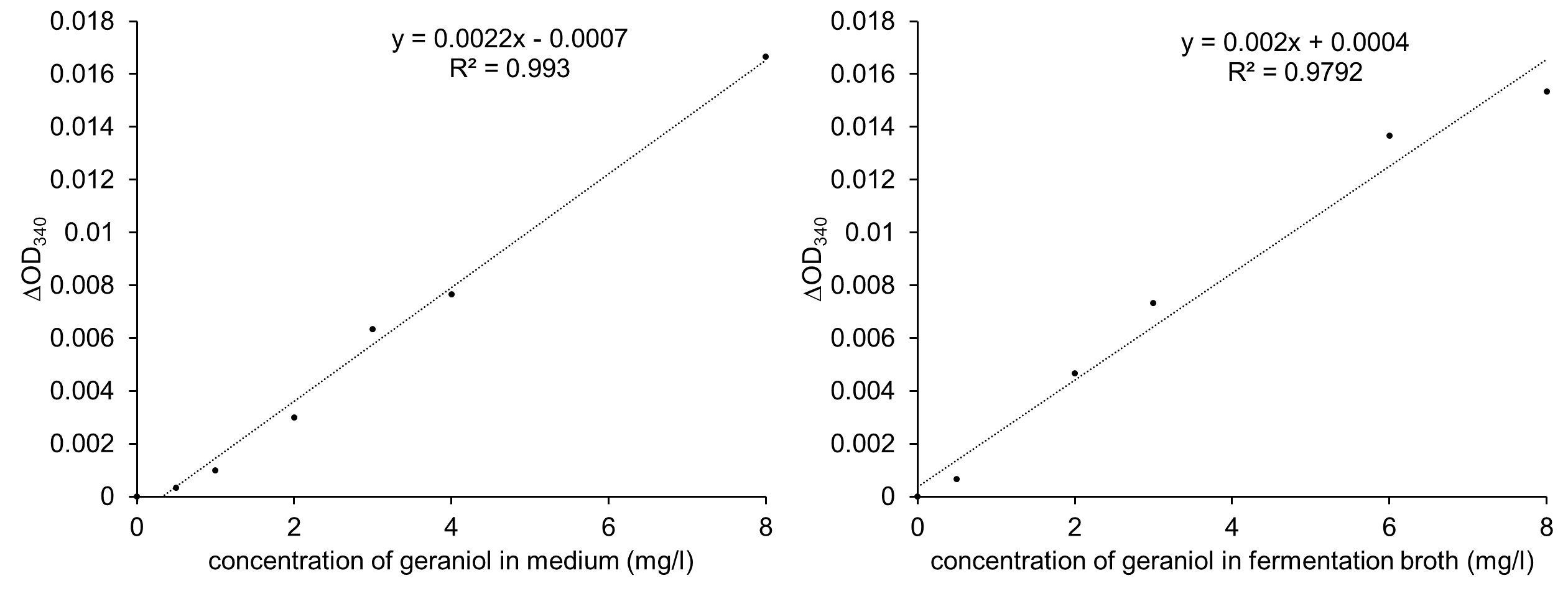


**Fig. S1.** The standard curve of geraniol reaction in medium or fermentation broth.

**
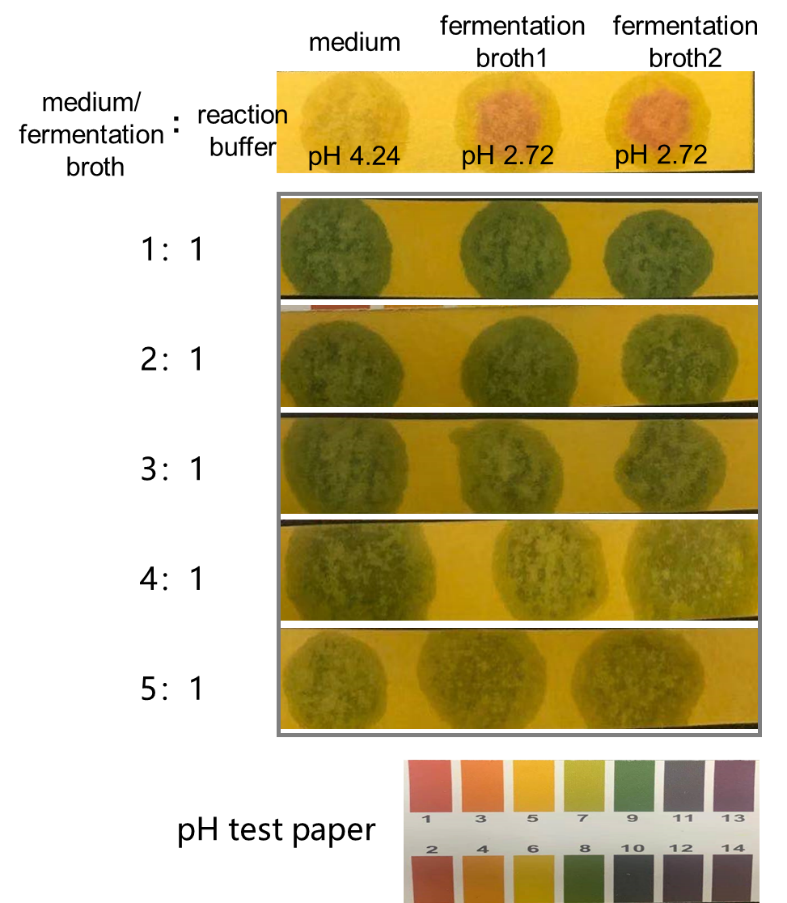
**

**Fig. S2.** Effect of fermentation broth on pH of reaction system. Compared with the pH test paper, the color fading means the pH value of reaction system decreased. Fermentation broth sample was set 2 repeats.


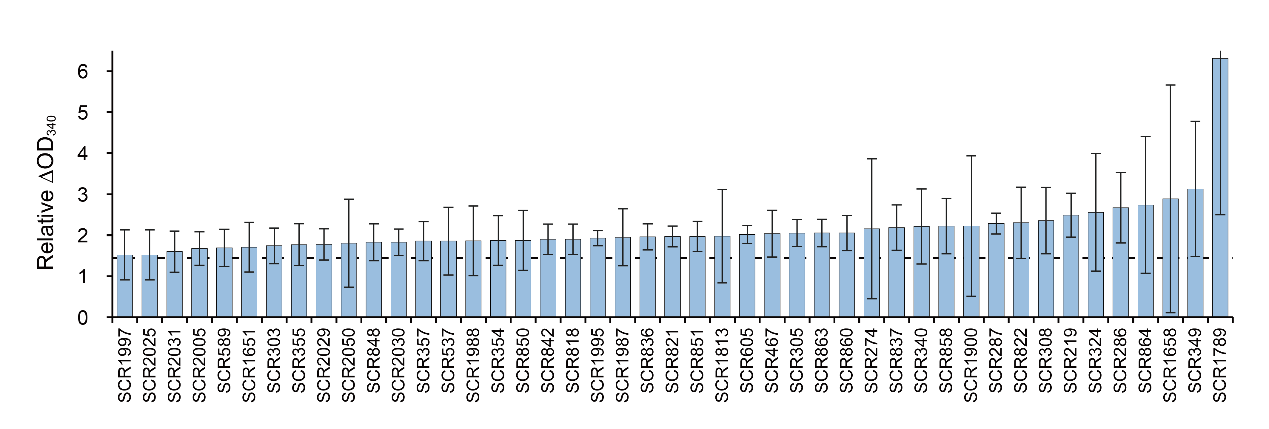


**Fig. S3**. The enzyme-coupling screening of geraniol high-yield SCRaMbLEd strains *in vitro* with detecting ∆OD_340_ by microplate reader. The relative ∆OD_340_=∆OD_340_ value of SCRaMbLEd strain/ ∆OD_340_ value of starting strain. The cutoff line was ∆OD_340_=1.5.


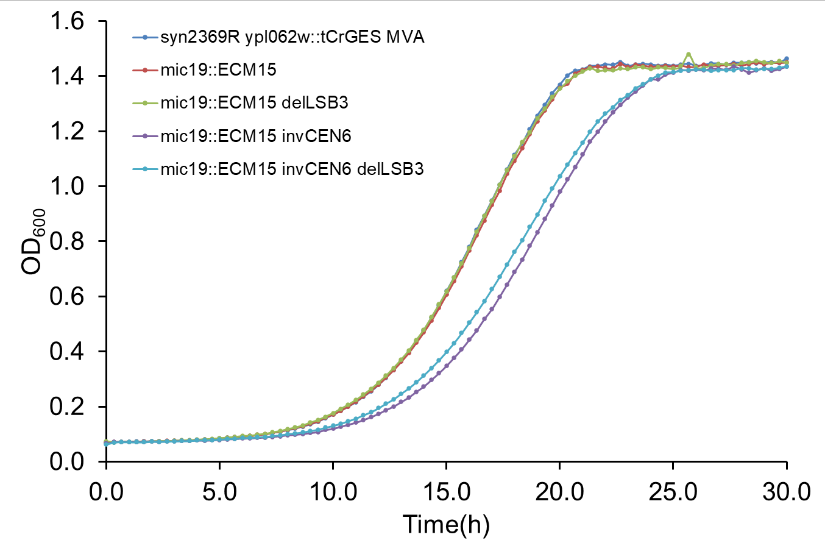


**Fig. S4** The growth curve of strains with potential synthesis regulatory targets combined.


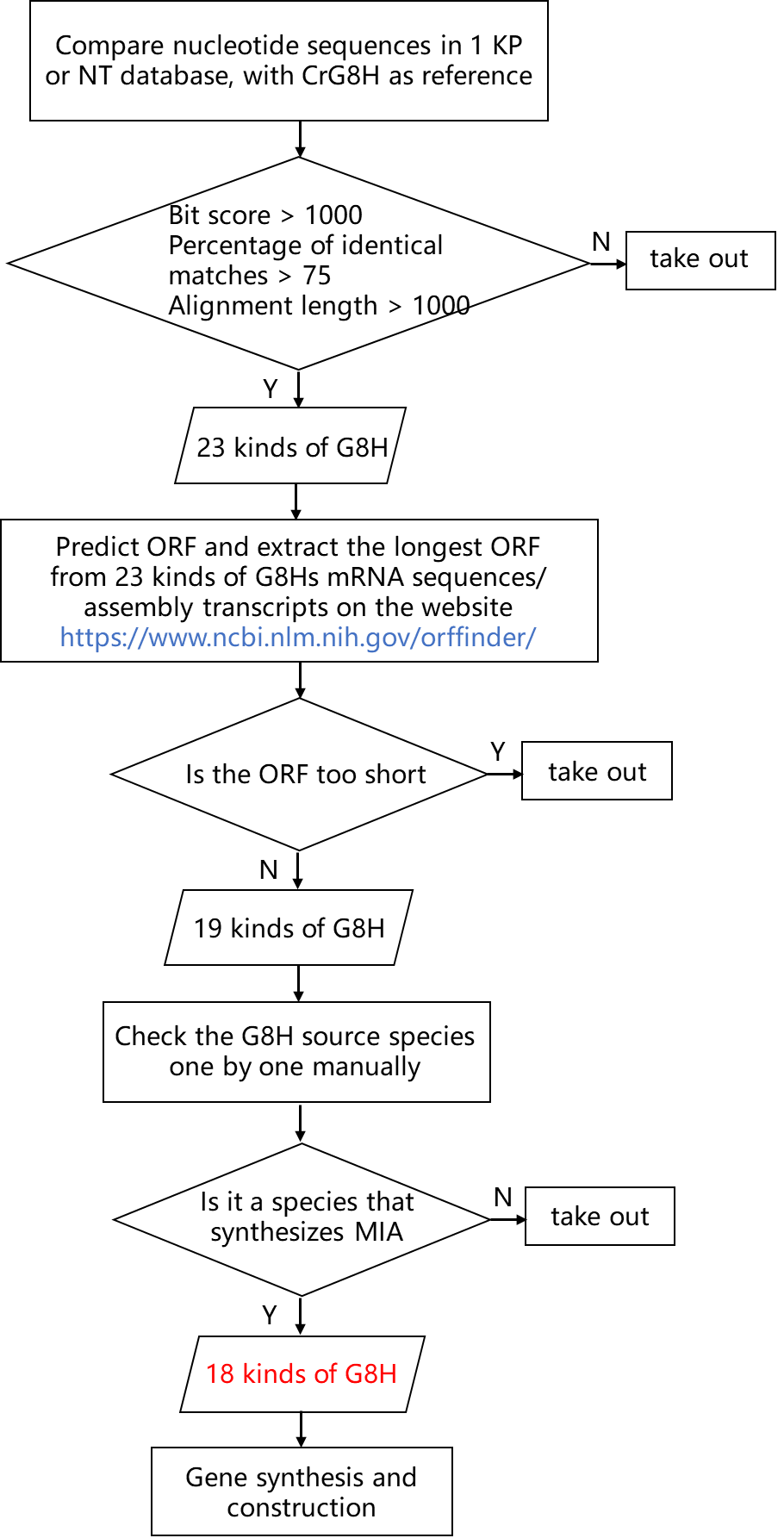


**Fig. S5.** Bioinformatics process for G8H homologous genes mining.


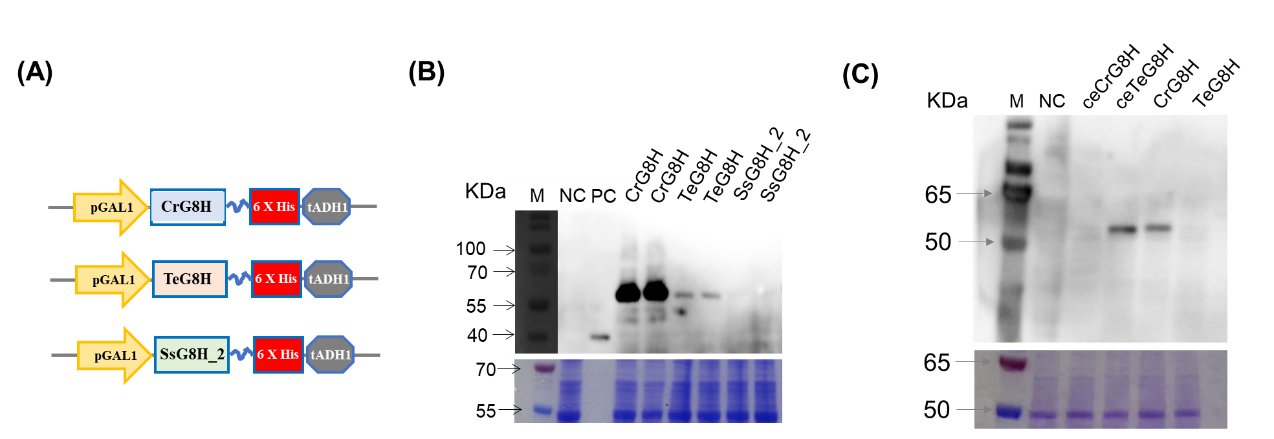


**Fig. S6.** Characterization of various G8Hs protein concentrations in yeast cell lysate. **(A)**. Schematic diagram of constructing three G8H fusion proteins with 6 histidine residues (6ⅹHis tag). **(B)**. SDS-PAGE electrophoresis (below) and Western blot (above) of three G8H proteins in yeast cell lysate. NC is cell lysate of control strain (ypl062w::tCrGES MVA); PC is a purified protein that can bind to the same antibody. 2 biological replicates per sample. **(C)**. SDS-PAGE electrophoresis (below) and Western blot (above) of two chimeric enzymes ceCrG8H and ceTeG8H in yeast cell lysate. NC is cell lysate of control strain (ypl062w::tCrGES MVA).


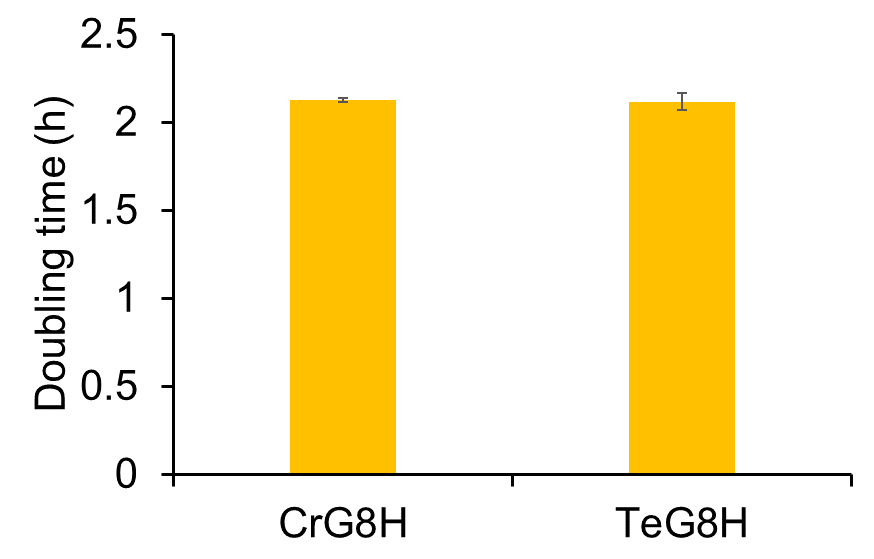


**Fig. S7.** Doubling time of 8-hydroxygeraniol producing strains with high expression enzyme (CrG8H) or low expression enzyme (TeG8H). Error bars show the mean ± standard error of three biological replicates.


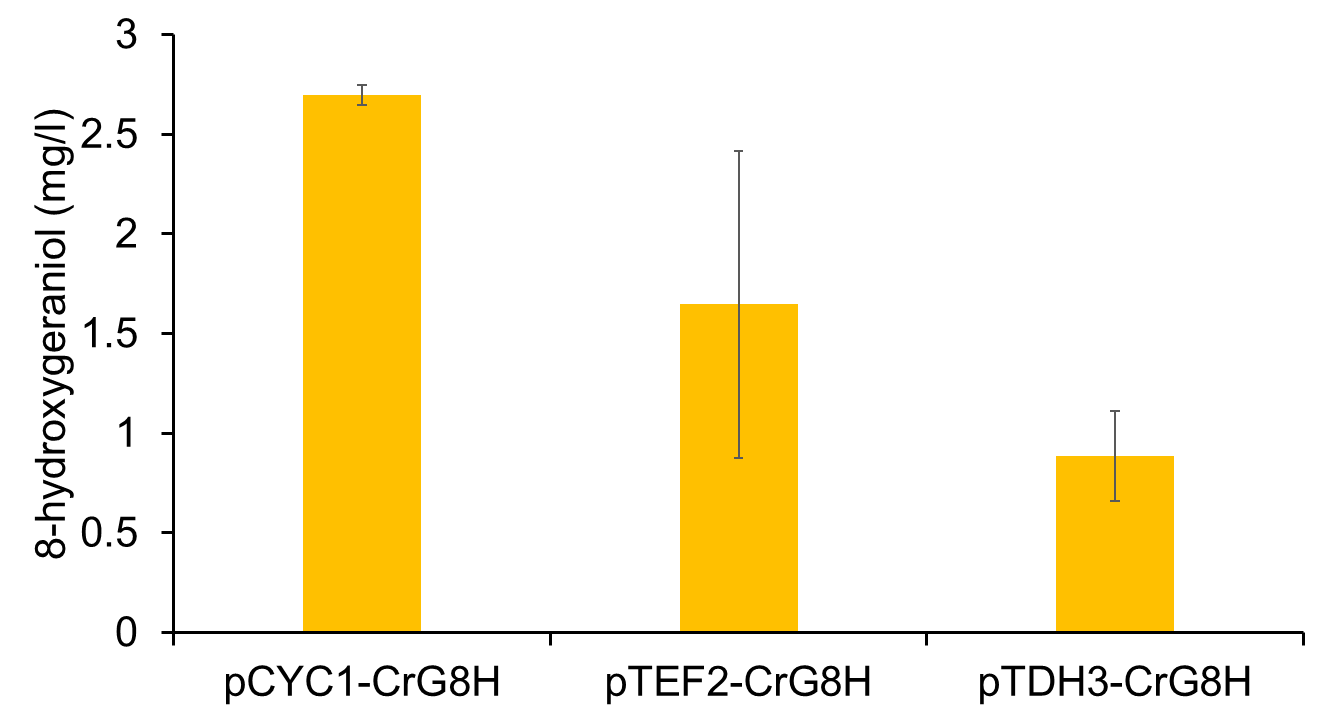


**Fig. S8.** The 8-hydroxygeraniol yield of strains with CrG8H driven by different strength of promoters. Error bars show the mean ± standard error of three biological replicates.

**References**

1. Campbell A, Bauchart P, Gold ND, Zhu Y, De Luca V, Martin VJ. Engineering of a nepetalactol-producing platform strain of Saccharomyces cerevisiae for the production of plant seco-iridoids. *ACS Synth Biol*. 2016;5:405-414.

2. Yee DA, DeNicola AB, Billingsley JM, Creso JG, Subrahmanyam V, Tang Y. Engineered mitochondrial production of monoterpenes in Saccharomyces cerevisiae. *Metab Eng*. 2019;55:76-84.

3. Dusséaux S, Wajn WT, Liu Y, Ignea C, Kampranis SC. Transforming yeast peroxisomes into microfactories for the efficient production of high-value isoprenoids. *Proc Natl Acad Sci U S A*. 2020;117:31789-31799.

4. Davies ME, Tsyplenkov D, Martin VJ. Engineering Yeast for De Novo Synthesis of the Insect Repellent Nepetalactone. *ACS Synth Biol*. 2021;10:2896-2903.

5. Wang H, Jiang G, Liang N, et al. Systematic Engineering to Enhance 8-Hydroxygeraniol Production in Yeast. *J Agric Food Chem*. 2023;71:4319-4327.

6. Baker Brachmann C, Davies A, Cost GJ, et al. Designer deletion strains derived from Saccharomyces cerevisiae S288C: a useful set of strains and plasmids for PCR‐mediated gene disruption and other applications. *Yeast*. 1998;14:115-132.

7. Guo Y, Dong J, Zhou T, et al. YeastFab: the design and construction of standard biological parts for metabolic engineering in Saccharomyces cerevisiae. *Nucleic Acids Res*. 2015;43:e88.

8. Sikorski RS, Hieter P. A system of shuttle vectors and yeast host strains designed for efficient manipulation of DNA in Saccharomyces cerevisiae. *Genetics*. 1989;122:19-27.

9. Zhang Y, Chiu T, Zhang J, et al. Systematical Engineering of Synthetic Yeast for Enhanced Production of Lycopene. *Bioengineering*. 2021;8:14.

10. Dymond JS, Richardson SM, Coombes CE, et al. Synthetic chromosome arms function in yeast and generate phenotypic diversity by design. *Nature*. 2011;477:471-476.

11. Matasci N, Hung LH, Yan Z, et al. Data access for the 1,000 Plants (1KP) project. *Gigascience*. 2014;3:17.
